# *In Situ* Evaluation of Oxidative Stress in Rat Fatty Liver Induced by a Methionine- and Choline-Deficient Diet

**DOI:** 10.1155/2016/9307064

**Published:** 2016-01-06

**Authors:** Isabel Freitas, Eleonora Boncompagni, Eleonora Tarantola, Cristian Gruppi, Vittorio Bertone, Andrea Ferrigno, Gloria Milanesi, Rita Vaccarone, M. Enrica Tira, Mariapia Vairetti

**Affiliations:** ^1^Department of Biology and Biotechnology “Lazzaro Spallanzani”, University of Pavia, 27100 Pavia, Italy; ^2^Department of Molecular Medicine, University of Pavia, 27100 Pavia, Italy; ^3^Department of Internal Medicine and Therapeutics, University of Pavia, 27100 Pavia, Italy

## Abstract

Nonalcoholic fatty liver disease (NAFLD) is a serious health problem in developed countries. We documented the effects of feeding with a NAFLD-inducing, methionine- and choline-deficient (MCD) diet, for 1–4 weeks on rat liver oxidative stress, with respect to a control diet. Glycogen, neutral lipids, ROS, peroxidated proteins, and SOD2 were investigated using histochemical procedures; ATP, GSH, and TBARS concentrations were investigated by biochemical dosages, and SOD2 expression was investigated by Western Blotting. In the 4-week-diet period, glycogen stores decreased whereas lipid droplets, ROS, and peroxidated proteins expression (especially around lipid droplets of hepatocytes) increased. SOD2 immunostaining decreased in poorly steatotic hepatocytes but increased in the thin cytoplasm of macrosteatotic cells; a trend towards a quantitative decrease of SOD expression in homogenates occurred after 3 weeks. ATP and GSH values were significantly lower for rats fed with the MCD diet with respect to the controls. An increase of TBARS in the last period of the diet is in keeping with the high ROS production and low antioxidant defense; these TBARS may promote protein peroxidation around lipid droplets. Since these proteins play key roles in lipid mobilization, storage, and metabolism, this last information appears significant, as it points towards a previously misconsidered target of NAFLD-associated oxidative stress that might be responsible for lipid dysfunction.

## 1. Introduction 

Nonalcoholic fatty liver disease (NAFLD), the most recurrent liver disorder in Western countries, is commonly associated with obesity and progression of metabolic syndrome [1–6]. NAFLD comprehends a wide spectrum of disorders ranging from simple steatosis, that is, excessive accumulation of triglycerides in the liver exceeding 5% of total organ mass, to nonalcoholic steatohepatitis (NASH), characterized by progressive inflammation associated with oxidative stress and apoptosis, often progressing to fibrosis, cirrhosis, and even hepatocellular carcinoma.

For investigating the etiology and regulation of NAFLD and NASH and for testing potential therapeutic approaches, several experimental models were developed. These are based on the application of a diet, on the administration of drugs to laboratory animals, or on the exposure of hepatic cell lines to these drugs; genetically modified rodents or zebrafish have also been introduced [7–12]. Whereas genetic models do not spontaneously progress into NASH, diet-based models have the advantage of allowing the evaluation of the progression of NAFLD within relatively short periods. One of the most commonly used dietary models of NAFLD and NASH is the methionine- and choline-deficient (MCD) diet, capable of increasing both liver lipid accumulation and oxidative stress, the “two hits” requested for the pathogenesis of steatohepatitis [13].

The MCD diet contains high levels of sucrose and fat but lacks methionine and choline. Choline is an essential nutrient fundamental for cell membrane integrity, transmembrane signaling, phosphatidylcholine synthesis, neurotransmission, and methyl metabolism [14]. In particular, phosphatidylcholine is required for the export of triglycerides by very-low-density lipoproteins (VLDLs). The decreased VLDL secretion together with an increase of the uptake of fatty acids was shown to be an important mechanism by which the MCD diet promotes intrahepatic lipid accumulation [15]. Furthermore, a significant reduction of fatty acid *β*-oxidation was demonstrated in MCD diet fed rats compared to controls [16]. Methionine is an essential amino acid fundamental for protein synthesis and for the synthesis of two key antioxidant molecules, S-adenosylmethionine (SAM) and glutathione (GSH). When the production of Reactive Oxygen Species (ROS) exceeds the antioxidant capacity of hepatocytes, extensive cellular damage affects DNA and membrane phospholipids, contributing to cell death, tissue injury, chronic inflammatory responses, and fibrogenesis [17]. For this reason, the MCD diet induces severe liver injury that is histologically similar to human NASH [7, 8, 14, 18, 19].

A rich literature is available documenting oxidative stress associated with liver steatosis (e.g., [20, 21]). Oxidative stress was proposed to be the second hit leading to the development of steatohepatitis [5]. Most studies on oxidative stress in fatty liver are based on biochemical evaluation of parameters such as total glutathione (GSH) and thiobarbituric acid reactive substances (TBARS) or of antioxidant enzymes in tissue homogenates or in plasma. One disadvantage of biochemical analyses is that they are performed on homogenates that do not allow the contribution of the single cell types and the possible influence of metabolic zonation to be appraised. The liver indeed normally exhibits metabolic zonation, that is, heterogenous distribution of metabolic functions across the lobule; in particular, oxygen-dependent functions like gluconeogenesis, ureagenesis, and oxidative phosphorylation predominate in the periportal region, whereas glycolysis, lipogenesis, and xenobiotic metabolism take place predominantly in the pericentral (perivenous) region [22, 23].

Histochemistry provides a unique opportunity to document* in situ* the behavior of single cells in a complex organ [24]. The purpose of the present work was to document, mainly with histochemical procedures, the progression of oxidative stress in the liver of rats induced by a MCD diet lasting for 1 to 4 weeks, taking as reference the liver of rats submitted to a control diet, isocaloric with MCD diet, but containing methionine and choline. As main effectors of the oxidative stress, Reactive Oxygen Species (ROS) were demonstrated; as targets of the oxidative stress, lipid droplets were investigated; as indices of ROS-induced injury, carbonyl groups induced in proteins by oxidative stress were evidenced; and as a key component of the antioxidant defense, the expression of Mn-dependent Superoxide Dismutase 2 (SOD2) was investigated. These parameters were complemented by biochemical analyses of ATP, GSH, and thiobarbituric acid-positive substances.

Our approach successfully demonstrated a lobular-zone dependent evolution of ROS production and effects throughout the 4-week period of the diet. In particular, it revealed oxidative-induced alteration of proteins in the coat of lipid droplets in hepatocytes.

## 2. Materials and Methods

*Chemicals.* Unless otherwise stated, the reagents were of the highest purity grade available and were purchased from Sigma (Milan, Italy).

### 2.1. Animal Model

Male Wistar rats were fed with methionine- and choline-deficient (MCD) diet (Laboratorio Dottori Piccioni, Gessate, Italy) [25] for 1–4 weeks. As controls, rats fed with choline and methionine containing diet, isocaloric with MCD diet, were used. A total of 6 rats were considered for each week: one fed with a control diet and 5 with MCD diet (except for the 2nd week). Rats were anesthetised (Pentobarbital i.p. injection, 40 mg/kg) and sacrificed, and liver specimens were collected, immediately frozen in liquid nitrogen, and stored at −80°C until being processed for biochemical assays or histochemical assays on cryostat sections, 8 *μ*m thick, cut at −24°C on a Leica CM 1850 cryostat. Other specimens were fixed and embedded in Paraplast for further histochemical determinations, as described below.

The use of the animal model was approved by the Italian Ministry of Health and Pavia University Animal Care Commission.

### 2.2. Determination of Glycogen Stores

Tissue samples were fixed in 2% p-formaldehyde in 0.1 M phosphate buffer (pH 7.4) for 24 h, dehydrated, and included in Paraplast. Glycogen was visualized with the periodic acid-Schiff reaction on liver sections of 7 *μ*m (Leica RM 2125 microtome) dewaxed in xylene and rehydrated in alcohol.

### 2.3. Lipid Detection [26]

Frozen sections (8 *μ*M thick) were collected on glass slides and stored at −80°C until use. A stock solution of 0.5 mg of Nile Red (Eastman Kodak Co., Rochester, NY, USA) was prepared in 1 mL of acetone and was stored in a refrigerator in a container protected from light and watertight until use. A working solution at 0.5% was prepared by dissolving a 5 *μ*L stock solution in 1 mL of ultrapure glycerol (99%), degassed under vacuum, and stored at 4°C protected from light. The solution was removed from the refrigerator an hour before use directly on the slide. Slides were finally observed by fluorescence microscopy after 5 minutes.

The filter set 09 (BP450-490, FT510, and LP515), which includes an excitation filter BP 450–490 nm (blue light), a dichroic mirror 510 nm, and a barrier filter 520 nm, was used to excite and reveal Nile Red-induced yellow-gold fluorescence.

### 2.4. Reactive Oxygen Species

Frozen sections (8 *μ*m thick) were placed onto clean glass slides and used immediately for the ROS tests. The DAB-Mn^2+^-Co^2+^ technique was used [27]. Briefly, unfixed cryostat sections were incubated for 30 min at 37°C with a medium containing 12.5 mM DAB, 5 mM MnCl_2_, and 40 mM CoCl_2_ dissolved in 10% w/v PVA (average mol. wt. 70,000–100,000), in 100 mM Tris-maleate buffer (pH 8.0). After incubation, the sections were rinsed in hot distilled water (60°C) to stop the reaction immediately and to remove the viscous incubation medium. The sections were mounted in glycerol jelly. Since the colored product is light sensitive, the slides were kept in the dark at 4°C until observation.

### 2.5. Immunohistochemical Detection of Carbonyl Groups in Peroxidated Proteins Derivatized with Dinitrophenyl Hydrazine [28]

Frozen tissue sections (8 *μ*m thick) were dried at room temperature for 1 h and then fixed using a mixture of diethyl ether and ethanol (1 : 1, v/v) for 15 min, before being incubated in 0.3% of 2,4-dinitrophenylhydrazine (DNPH) dissolved in absolute ethanol containing 1.5% (v/v) sulfuric acid for 16 h, washed in absolute ethanol containing 1.5% (v/v) sulfuric acid for 5 min, and then rehydrated. Endogenous peroxidase activity was blocked with a solution of 3% H_2_O_2_ in 10% methanol for 20 min. After two washing instances in PBS for 5 min, nonspecific binding sites were blocked with 2.5% normal horse serum in PBS for 1 h. Further block was made with 5% skim milk and 1.5% NaCl in PBS for 30 min. The sections were then incubated with a rabbit monoclonal anti-dinitrophenyl (DNP) antibody (SP-0603; Vector Laboratories) diluted 1 : 100 in 2.5% normal horse serum at 4°C for 1 h. After two further 5 min washing instances in PBS, the sections were incubated with ImmPRESS HRP anti-rabbit Ig (peroxidase) Polymer Detection Kit (MP-7401; Vector Laboratories, Burlingame, CA 94010, USA) for 30 min at room temperature. Sections were washed twice in PBS for 5 min and incubated for 3 min with liquid DAB + chromogen system (K3468; DAKO, Carpinteria, California, USA). After 5 min washing in distilled water, the slides were mounted with glycerol gelatin. Control reactions were performed by replacing the primary antibody with PBS.

### 2.6. Immunohistochemical Demonstration of SOD2

Methacarn solution consisting of 60% (vol/vol) absolute methanol, 30% chloroform, and 10% glacial acetic acid was freshly prepared before fixation. Tissue samples were fixed in Methacarn overnight at 4°C. For embedding, tissue samples were dehydrated three times for one hour in fresh 100% ethanol at 4°C, immersed in xylene once for one hour and then thrice for 1 h at room temperature, and embedded in Paraplast. Paraplast-embedded sections (6 *μ*m thick) were cut, dewaxed in xylene, and rehydrated with graded ethanol and water. Antigen retrieval was performed by microwave treatment with an Antigen Unmasking Solution (Vector Laboratories, Inc., Burlingame, CA) with 2 cycles of 5 min at 750 W. After cooling for 20 min at room temperature, two washing instances in PBS for 5 min were done. Endogenous peroxidase activity blocking was performed with 10% methanol-3% H_2_O_2_ in PBS for 20 min. After washing twice for 5 min in PBS, nonspecific site blocking was performed with 2.5% normal horse serum in PBS for 1 h. The sections were then incubated with a primary rabbit polyclonal antibody against SOD2 (NB100-1992; Novus Biologicals, Littleton, CO 80120, USA) diluted 1 : 300 in 2.5% normal horse serum at room temperature for 1 h. After two further 5 min washing instances in PBS, the sections were incubated with ImmPRESS HRP Universal Antibody (anti-mouse Ig/anti-rabbit Ig, peroxidase) Polymer Detection Kit (MP-7500; Vector Laboratories, Burlingame, CA 94010, USA) for 30 min at room temperature. Sections were washed twice in PBS for 5 min and incubated for 3 min with liquid DAB + chromogen system (K3468; DAKO, Carpinteria, California, USA). After 5 min washing in distilled water, sections were counterstained with Hematoxylin for 5 min, washed in tap water for 10 min and in distilled water for 2 min, dehydrated, and mounted with Entellan (Merck, Whitehouse Station, NJ, USA). Control reactions were performed by replacing the primary antibody with PBS.

### 2.7. Microscopy and Photomicrography of Histochemical Reactions

The slides were observed with a Zeiss Axioskop 2 Plus light microscope (Carl Zeiss Microimaging, Jena, Germany) equipped with Differential Interference Contrast (DIC) and with the 09 Zeiss filter set to demonstrate Nile Red-induced yellow-gold fluorescence of neutral lipids: BP 450–490 excitation filter, FT 510 beam splitter, and LP515 emission filter. Photomicrography was made using a Canon EOS 1100D digital camera (Tokyo, Japan) set to a resolution of 6 megapixels.

### 2.8. Biochemical Assays

Tissue ATP was measured by the luciferin-luciferase method with the ATP Bioluminescence Assay Kit CLS II (Roche Molecular Biochemicals, Milan, Italy). The hepatic concentration of reduced glutathione (GSH) was measured by an enzymatic method (Cayman Chemical Co., Ann Arbor, MI, USA). Hepatic lipid peroxidation was assessed by measuring the amount of thiobarbituric acid reactive substances (TBARS) formation, which was measured according to the method of Esterbauer et al. [29]. The TBARS concentrations were calculated using malondialdehyde (MDA) as standard. Tissue protein content was assayed by the method of Lowry et al. [30].

Statistical analysis of data concerning ATP, GSH, and TBARS concentration was performed using R software (R Development Core Team). Data normally distributed were analyzed by one-way ANOVA, followed by Tukey's multiple comparisons test. Data not normally distributed were analyzed by Kruskal-Wallis test, followed by Dunn's multiple comparisons test. The value of *P* < 0.05 was considered to indicate statistical significance. Graphs present the mean value ± standard error of the mean (SEM).

### 2.9. Western Blot Assessment of SOD2 Expression

40 mg of liver was lysed in 1 mL of RIPA buffer (50 mM Tris-HCl, 1% NP-40, 0.25% sodium deoxycholate, 150 mM NaCl, and 1 mM Na_3_VO_4_, pH 7.4) supplemented with Protease Inhibitor Cocktail. Lysates were then centrifuged for 10 min at 4°C, avoiding rescuing the lipids ring in the samples. Protein concentration was measured with a spectrophotometer (UV-1601, Shimadzu, Tokyo, Japan) set to 562 nm, using Pierce BCA Protein Assay Kit (bicinchoninic acid, Thermo Fisher Scientific Inc., Rockford, IL, USA). Sample buffer (3x) consisting of 6% SDS, 1.5% DTT, 30% glycerol, 0.03% bromophenol blue, and 0.5 M Tris-Gly buffer (1x, containing SDS 0.1%, pH 8.3) was added to the appropriate aliquots of supernatants. After boiling for 10 min at 95°C, equal aliquots of protein were separated by 12% SDS-PAGE under standard conditions, followed by wet transfer onto PVDF membrane (Bio-Rad Laboratories, Hercules, CA, USA) at 4°C for 2 h. After blocking with 0.1% Tween-20-TBS containing 5% BSA for 1 h at room temperature, membranes were incubated overnight with rabbit polyclonal anti-SOD2 primary antibody (NB100-1992; Novus Biologicals, Littleton, CO 80120, USA) diluted 1 : 2000 and, at a later stage, with a monoclonal anti-*β*-actin primary antibody (#A1978, clone AC-15, Sigma-Aldrich) diluted 1 : 5000 in the blocking solution. Membranes were then washed with PBS containing Tween-20 at 0.5% at room temperature and then incubated with a corresponding HRP-conjugated secondary antibody diluted 1 : 3000 for 1 h at room temperature. After new washing instances at room temperature, the bands were revealed by incubation of the membranes with Millipore Immobilon Western Chemiluminescent HRP Substrate [SuperSignal West Dura (Pierce, Thermo Fisher Scientific Inc.)] for 5 min and then visualized by ChemiDoc XRS+ System (Bio-Rad Laboratories), to get digital images.

Digital images were analyzed using Quantity One 1-D Analysis Software, Bio-Rad Laboratories. The optical density (OD) of each band was calculated following the manufacturer's instructions. The OD values of SOD2 were normalized versus own relative band of *β*-actin for each sample.

## 3. Results

### 3.1. Histochemical Analyses of Control Liver

The liver of rats fed, for two weeks, with a diet isocaloric with the MCD diet but containing methionine and choline was considered representative and herewith described as “control.”

#### 3.1.1. Glycogen Stores

**Figure 1:**
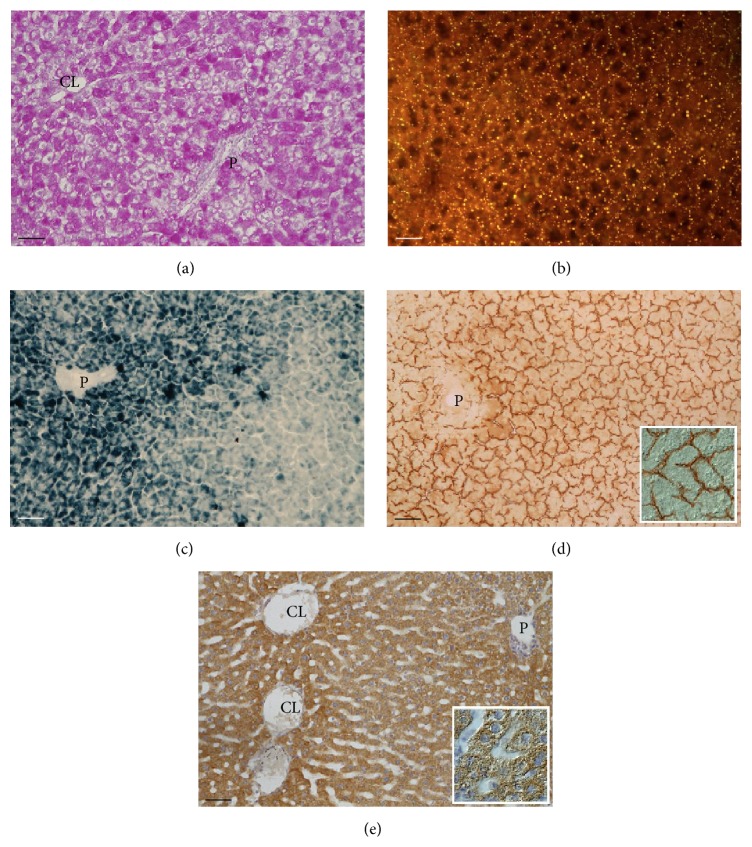
Liver of rats submitted to control diet for 2 weeks. (a) Representative photomicrographs of PAS reaction for glycogen, abundant in all hepatocytes. (b) Nile Red-induced fluorescence of small neutral lipid droplets in the perisinusoidal cytoplasm of hepatocytes. (c) Diaminobenzidine-Mn^2+^-Co^2+^ reaction for Reactive Oxygen Species (ROS); intense reaction in periportal hepatocytes and occasional Kupffer cells in the midzone or pericentral regions. (d) Immunoreaction against dinitrophenyl (DNP) groups for demonstrating carbonyl groups derivatized with dinitrophenyl hydrazine (DNPH); these are present in perisinusoidal and canalicular membrane domains of hepatocytes. (e) Immunoreaction for visualizing the expression of Mn-dependent Superoxide Dismutase 2 (SOD2); moderately intense staining in the cytoplasm of periportal and pericentral hepatocytes. P: branch of portal vein; CL: branch of centrolobular vein. Scale bar: 50 *μ*m (insets in (d) and (e) represent image details at higher magnification).

Glycogen deposits, evidenced by a fuchsia precipitate with the PAS reaction, were present in hepatocytes throughout the lobule; they were particularly abundant in periportal and centrolobular cells (Figure 1(a)).

#### 3.1.2. Neutral Lipid

Small neutral lipid droplets, evidenced by the yellow-gold fluorescence induced by Nile Red, were present in all hepatocytes; these were polarized towards the sinusoidal domains (Figure 1(b)).

#### 3.1.3. Reactive Oxygen Species (ROS)

Lobular zonation of ROS, demonstrated by a blue precipitate with the Mn-DAB-Co reaction, was observed. The reaction was particularly intense in the cytoplasm of hepatocytes in the periportal/midzone region, where it was polarized towards the sinusoidal domains, and very low in the pericentral region. Occasional sinusoidal cells displayed strong staining (Figure 1(c)).

#### 3.1.4. Carbonyl Groups Derivatized with 2,4-Dinitrophenylhydrazine (DNPH)

Resulting dinitrophenyl (DNP) groups were demonstrated with an immunoperoxidase method giving a brown final reaction product. Intense immunoreactivity was seen in sinusoidal and canalicular membrane domains of hepatocytes; diffuse lighter staining was also seen in the cytoplasm of periportal cells (Figure 1(d)).

#### 3.1.5. Superoxide Dismutase 2

Immunoreactivity for SOD2 was present in hepatocytes throughout the lobule, being intense in periportal and pericentral cells and moderate in hepatocytes of the midzone; it was present as a granular brown product in the cytoplasm partially polarized towards sinusoidal and canalicular domains (Figure 1(e)).

### 3.2. Histochemical Analyses of the Liver of Rats Fed with a MCD Diet for 1–4 Weeks

#### 3.2.1. Glycogen and Lipid Stores

**Figure 2:**
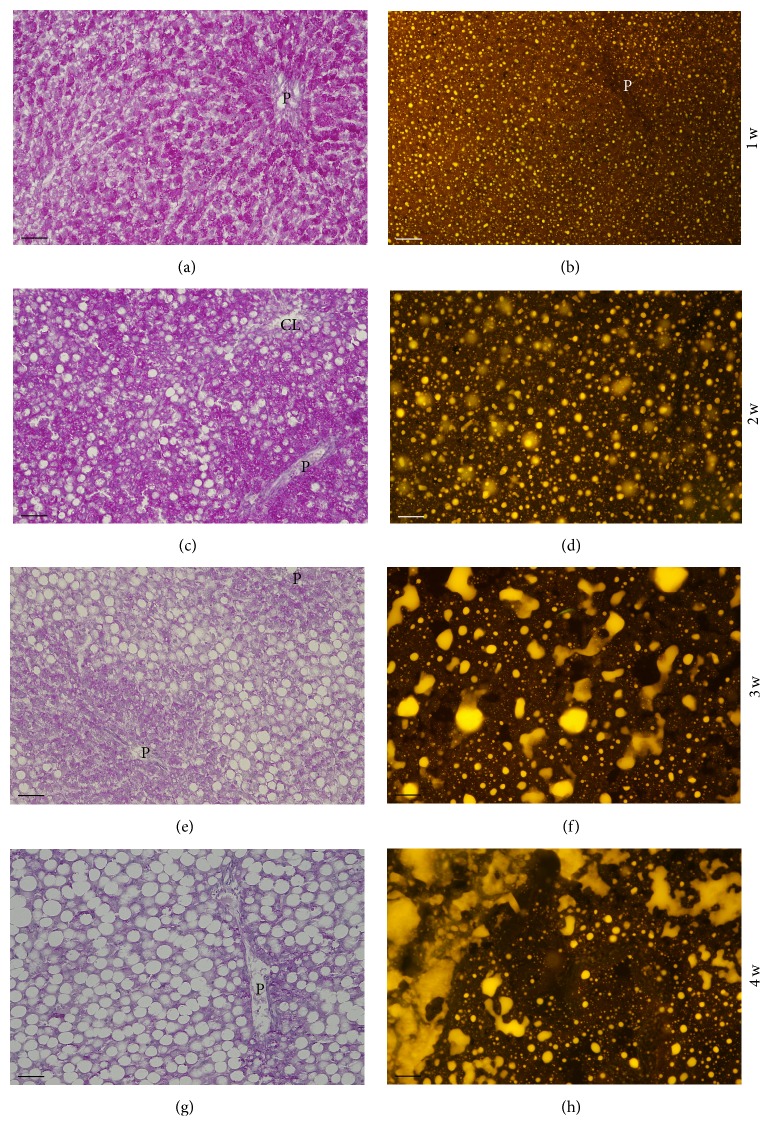
Comparison between glycogen accumulation (PAS reaction; (a), (c), (e), and (g)) and neutral lipid accumulation (Nile Red reaction; (b), (d), (f), and (h)) in the liver of rats fed with the MCD diet for 1–4 weeks. Representative photomicrographs. It is shown that whereas the glycogen content of the liver sharply decreases in the 2nd week of the diet and vanishes altogether in the 3rd and 4th weeks, the neutral lipid content dramatically increases from the 2nd to the 4th week. P: branch of portal vein; CL: branch of centrolobular vein. Scale bar: 50 *μ*m.

Figure 2 illustrates representative images of the change in liver stores of glycogen (evidenced with the PAS reaction) and of neutral lipid (fluorochromized with Nile Red), along the 4-week period of the MCD diet. Glycogen, abundant in animals fed with the MCD diet for 1 week, sharply decreased as the degree of steatosis increased, being rare in the 3rd week and almost absent in the 4th week (Figures 2(a), 2(c), 2(e), and 2(g)). Small-to-medium-sized neutral lipid droplets displaying bright-yellow fluorescence were present throughout the cytoplasm of hepatocytes already at the 1st week of diet (Figure 2(b)). With the progression of the diet, the dimension of the yellow-emitting droplets increased. In the 3rd and 4th weeks, the lipid droplets in these unfixed unfrozen sections coalesced forming complex and extended structures that impaired the visualization of details of single hepatocytes (Figures 2(d), 2(f), and 2(h)).

#### 3.2.2. Reactive Oxygen Species (ROS) and Carbonyl Groups Derivatized with 2,4-Dinitrophenylhydrazine (DNPH)

**Figure 3:**
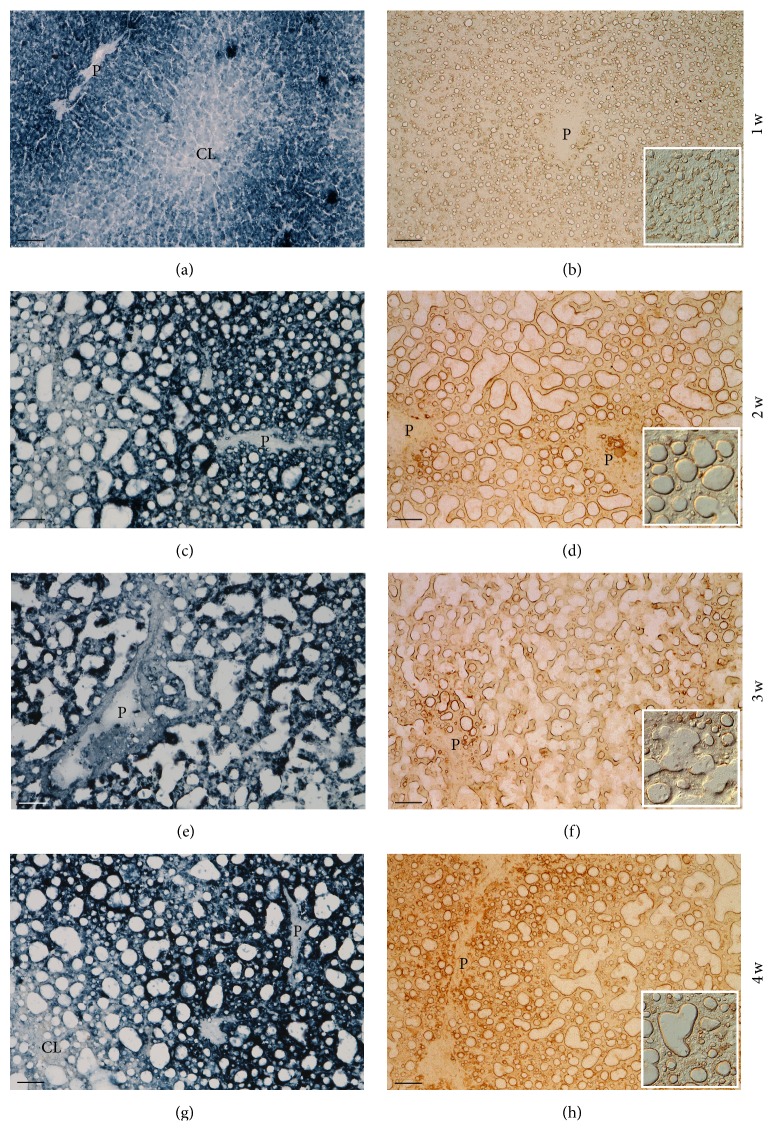
Comparison between ROS formation (DAB-Mn-Co reaction; (a), (c), (e), and (g)) and carbonyl groups derivatized with DNPH (immunoreaction against DNP; (b), (d), (f), and (h)) in the liver of rats fed with the MCD diet for 1–4 weeks. Representative photomicrographs. Whereas in the 1st week of the diet the lobular ROS pattern is similar to the control liver, from the 2nd week onwards, ROS formation is intense in all hepatocytes, being concentrated in the cytoplasm surrounding large lipid droplets. The patterns of peroxidized proteins follow the ROS trend. P: branch of portal vein; CL: branch of centrolobular vein. Scale bar: 50 *μ*m (insets: details at higher magnification).

Figure 3 shows representative images of the change in ROS production and carbonyl groups in the liver along the 4-week period of the MCD diet; both types of reaction were performed on frozen sections. The ROS pattern in the 2nd week of MCD diet (Figure 3(a)) closely resembles the control pattern in staining intensity and intralobular distribution of the colored product in hepatocytes and occasional sinusoidal cells; the parenchymata looked normal and hepatocyte contours discernable (Figure 1(c)). In the 2nd week of diet, large lipid droplets deformed hepatocytes. With respect to the 1st week, the ROS reaction was enhanced in the cytoplasm of steatotic hepatocytes, being concentrated mainly around lipid droplets (Figure 3(c)). In the 3rd and 4th weeks, the same trend was seen (Figures 3(e) and 3(g)). As far as derivatized carbonyl groups are concerned, in the 1st week of MCD diet, a low canalicular immunoreaction was observed only in the periportal region; a high reaction was instead present around lipid droplets (Figure 3(b)). On the 2nd week of diet (Figure 3(d)), the immunoreaction in hepatocyte membrane domains was no longer present whereas the larger lipid droplets were highly stained peripherally; microsteatotic hepatocytes showed moderate staining in the cytoplasm. Several strongly stained stromal cells were also present. Immunoreactive material was present in the lumen of veins. On the 3rd week of diet (Figure 3(f)), the immunoreaction surrounding large lipid droplets was still intense but the diffuse cytoplasmic immunoreaction in hepatocytes was lighter; immunoreactive stromal cells were abundant. On the 4th week (Figure 3(h)), the immunoreaction around the large lipid droplets was as strong as in the previous weeks, the diffuse reaction in the cytoplasm of periportal hepatocytes was again present, being even stronger than in the 2nd week, and positive stromal cells were abundant.

#### 3.2.3. Immunohistochemistry of Superoxide Dismutase 2 Expression

**Figure 4:**
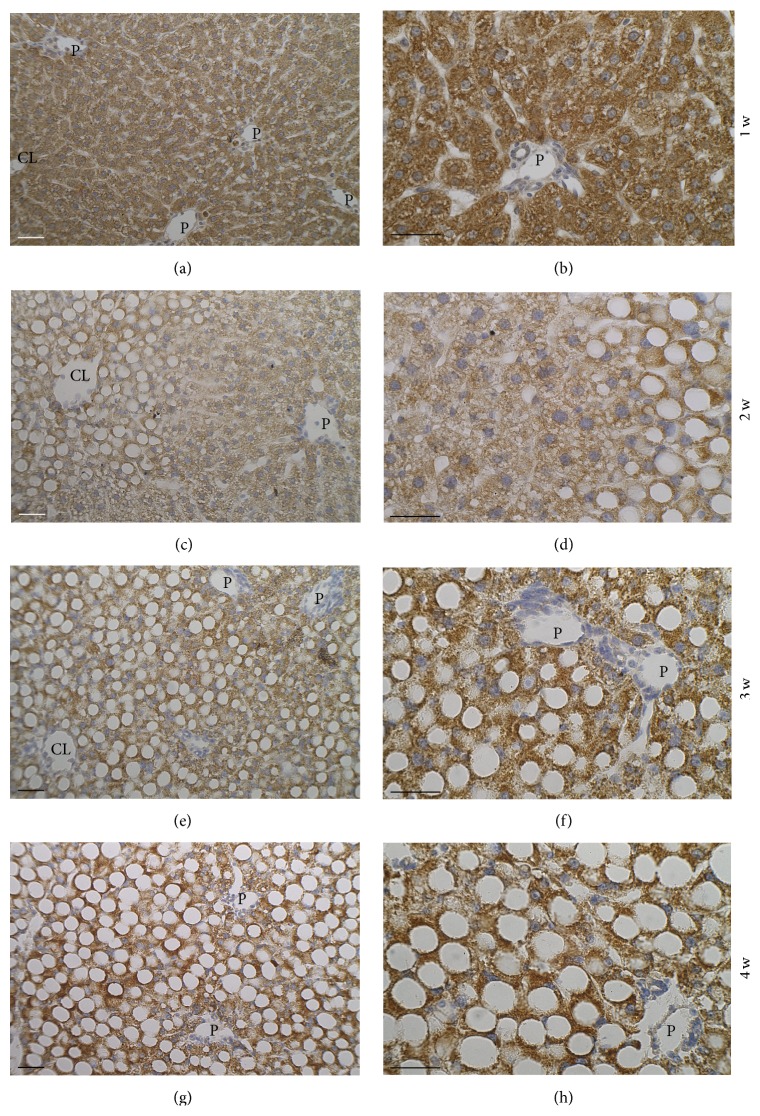
Expression of Mn-dependent Superoxide Dismutase 2 (SOD2) in the liver of rats fed with the MCD diet for 1–4 weeks. Representative photomicrographs. Immunoreactivity is concentrated in the thin rim of cytoplasm surrounding the large lipid droplets. P: branch of portal vein; CL: branch of centrolobular vein. Scale bar: 50 *μ*m.

Figure 4 shows representative images illustrating the changes in immunoreactivity for SOD2 in the liver during the 4-week period of MCD diet. In the 1st week, the reaction in hepatocytes was present throughout the parenchyma, with no apparent zonation (Figure 4(a)); higher magnification allows seeing that the granular product is concentrated in the cytoplasm surrounding the small lipid droplets that are negative for the reaction (Figure 4(b)). In the 2nd week, immunoreactivity of periportal and midzone hepatocytes is distributed around small lipid droplets in the abundant cytoplasm, whereas in pericentral, highly steatotic hepatocytes, the reaction is very intense and concentrated in the thin rim of cytoplasm surrounding the lipid droplets (Figures 4(c) and 4(d)). In the 3rd week (Figures 4(e) and 4(f)), the whole parenchyma is macrosteatotic and immunoreactivity is similar to that displayed by centrolobular cells in the 2nd week. In the 4th week, the dimension of the lipid droplets is apparently higher with respect to the 3rd week, further reducing the area of positive cytoplasm rim (Figures 4(g) and 4(h)).

#### 3.2.4. Superoxide Dismutase 2 Protein Levels Evaluated by Western Blot

**Figure 5:**
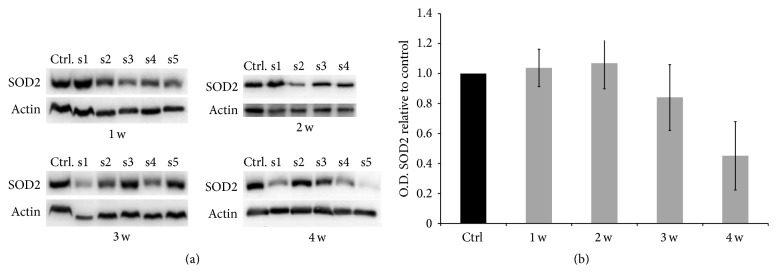
Evaluation of SOD2 expression in livers of the rats fed with control or MCD diet for 1–4 weeks. (a) Comparison of SOD2 expression in the liver of rats fed with control and MCD diet for 1–4 weeks. Histograms representing the ratio between optical density (OD) values of homogenates of the liver of rats fed with the MCD diet for 1 to 4 weeks and the OD of control livers (b).

Figure 5(a) shows Western blots for SOD2 expression and relative *β*-actin bands as equal loading of liver homogenates from rats fed with control diet or MCD diet for 1–4 weeks; Figure 5(b) shows SOD2 optical densities for rats fed with MCD diet for 1–4 weeks (normalized to control *β*-actin OD) with respect to control. A trend towards a decrease of SOD2 expression in the last weeks is detected.

#### 3.2.5. Biochemical Evaluation of ATP Content, Thiobarbituric Acid Reactive Substances, and Glutathione in the Liver of Control and MCD Diet Submitted Rats

**Figure 6:**
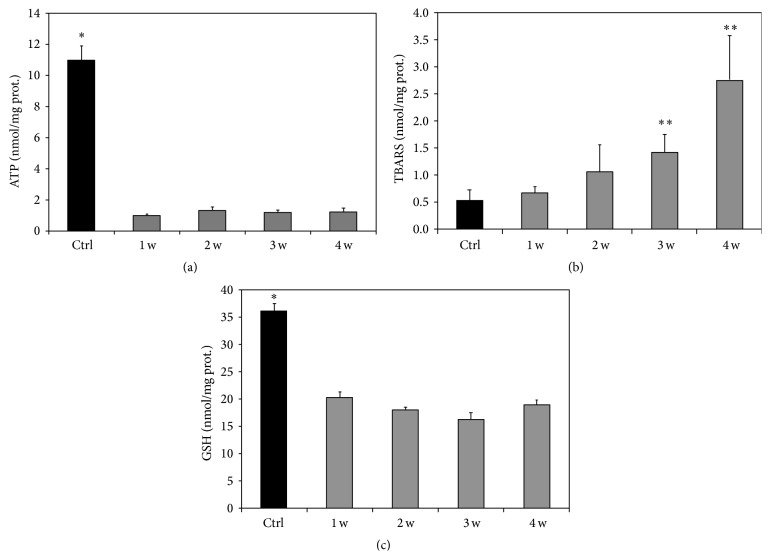
Changes in tissue ATP, TBARS, and GSH concentration in rats fed with a MCD diet with respect to values concerning a control diet. The results are reported as the mean ± SE. MCD diet groups versus control group. ^*∗*^ *P* < 0.05, ^*∗∗*^ *P* < 0.01.

A marked decrease in the hepatic levels of ATP and GSH was found already at the 1st week in rats fed with a MCD diet as compared with the control group (Figures 6(a) and 6(b)). The ATP and GSH content remained low up to the 4th week. By contrast, TBARS levels progressively increased in livers obtained from MCD-fed rats; the TBARS formation was significantly different from that of control livers after the 3rd week of MCD diet (Figure 6(c)).

## 4. Discussion

### 4.1. Control Livers

#### 4.1.1. Glycogen

The high content of glycogen in hepatocytes is in keeping with the high caloric diet (45% sucrose) to which the animals were submitted.

#### 4.1.2. Neutral Lipids

The droplets emitting bright-yellow fluorescence are ascribed to intracellular triacylglycerols (TAGs) depots and/or to lipoproteins [31, 32]. Lipid droplets (LDs), now considered as true organelles, are composed of a core of TAGs and other neutral lipids and surrounded by a monolayer of phospholipids [32]. LDs are coated with proteins such as perilipin, adipophilin, and tail-interacting protein of 47 kDa (TIP47), also referred to as the PAT proteins [33]. The proteins coating LDs are not only structural polypeptides and chaperones, but also enzymes with different key cellular functions such as TAG synthesis and ROS detoxification [33]. However, the small dimension of the droplets (microsteatosis) and their perisinusoidal location suggest these are mainly lipoproteins ready to be secreted into the sinusoidal circulation to deliver triglycerides and cholesterol to peripheral tissues.

#### 4.1.3. ROS

The histochemical reaction used is based on the ROS-induced oxidation of Mn^2+^ to Mn^3+^, which in turn oxidizes DAB, causing its polymerization; intensification of the colored polymer is achieved by Co^3+^ ions. This reaction proved to demonstrate mainly superoxide anion (O_2_^−^) and singlet oxygen (O_2_(^1^ΔG)) [27]. The strong ROS reaction in hepatocytes in the periportal and midzone regions is in keeping with the literature [27, 34]. Approximately, 2–5% of consumed oxygen normally undergoes one-electron reduction with the formation of O_2_^−^, mainly at the level of complex I (NADH coenzyme Q reductase) and complex III (ubiquinol cytochrome c reductase) [35]. The lobular zonation of ROS production is consistent with oxidative energy metabolism predominating in the periportal zone where oxygen tension is much higher than in the pericentral region [22]. The zonation of the colored product towards the perisinusoidal domain reflects the abundance of mitochondria in this region of the cytoplasm [36]. The reaction in sinusoidal cells indicates ROS production by NADPH oxidase in phagocytic cells (Kupffer and sinusoidal endothelial cells) [37].

#### 4.1.4. Carbonyl Groups Derivatized with 2,4-Dinitrophenylhydrazine (DNPH)

Protein carbonyl (CO) groups are considered as early biomarkers of oxidative stress [38, 39]. Most current assays for the detection of CO groups in oxidized proteins are based on the derivatization of the carbonyl group with DNPH, which leads to the formation of a dinitrophenyl (DNP) hydrazine product that can be recognized by specific antibodies [38, 39]. The strong reactivity observed in sinusoidal and canalicular domains of hepatocytes in the liver of control animals, not present when the immunoreaction was performed in the absence of the primary antibody, suggests that this procedure demonstrates carbonyl groups normally present in biomolecules as well. The glycocalyx of hepatocytes, identified with lectin affinity techniques, was shown to be particularly abundant in sinusoidal and canalicular domains [40]. Carbonyl groups of sialic acids of plasma membrane glycoproteins/glycolipids are likely candidates. Indeed, staining liver sections with the lectin* Sambucus nigra agglutinin *(SNA) which recognizes sialic acid linked *α*(2-6) to galactose revealed exclusive staining in canalicular membranes [41]. Furthermore, bile canaliculi staining was seen in mouse and rat liver with a chemical reaction selective for sialic acid involving a thiocarbohydrazide-silver protein-physical development (TCH-SP-PD) procedure [42]. Therefore, the immunostaining of the sinusoidal domain of hepatocytes must be due to other biomolecule(s). The lighter diffuse staining in the cytoplasm of periportal hepatocytes is ascribed to soluble biomolecules bearing carbonyl groups (e.g., pyruvate, glyceraldehyde-3-phosphate).

#### 4.1.5. Superoxide Dismutase 2

Superoxide Dismutases are key enzymes, which protect cells from oxidative stress by scavenging superoxide radicals through the dismutation into O_2_ and H_2_O_2_ [43, 44]. The enzyme considered in this study, Mn-SOD, is located mainly in the matrix of mitochondria [43, 44]. The expression of the mitochondrial isoform of SOD in hepatocytes throughout the liver lobule reflects the abundance of mitochondria in liver cells [45]. The high expression in periportal cells is in keeping with oxidative energy metabolism predominating in the periportal region, as mentioned above. Also, as above said, the zonation of the immunoprecipitate towards the perisinusoidal domain reflects the abundance of mitochondria in this region [36]. In the periportal and midzone regions, therefore, the intensity of the immunoreaction for SOD2 is in keeping with the high reactivity for ROS in the same area. However, no such correlation was seen between immunoreaction pattern for SOD2 and the ROS reaction in the pericentral region. The high expression of SOD2 might be related to the fact that pericentral hepatocytes contain high concentrations of oxidases engaged in xenobiotics detoxification, in particular P450 isoenzymes [46, 47]. In other words, in addition to its efficient catalysis of the dismutation of superoxide, Mn-SOD is presumed to act also as a nonspecific peroxidase, as shown for the cytosolic form, Cu, Zn-SOD [48]. SOD activity in pericentral hepatocytes would be particularly necessary since the most important nonenzymatic agent for ROS detoxification, glutathione (GSH), and enzymes that utilize this thiol, which are enriched in periportal hepatocytes, show very low expression in pericentral cells [44].

### 4.2. MCD Diet-Induced Changes

The progressive increase of neutral lipid droplets along the 4-week period of the MCD diet, visualized by the yellow-gold fluorescence of Nile Red, confirms the efficacy of the diet in inducing steatosis. The fluorochrome Nile Red has been used to document steatosis in rats fed with a diet rich in fructose and alcohol [49], with lipid droplets being visualized by green fluorescence; in that study, fluorescence emission was indeed detected with a band-pass emission filter centered at 590 nm that allows visualization of green emission only. In our case, we used a long-pass emission filter that allows detecting emissions longer than 515 nm, that is, from the green to the red regions of the spectrum. The accumulation of triglycerides in the cytoplasm of hepatocytes causing steatosis is considered the first of the two hits necessary for the induction of NAFLD [13, 50].

In parallel with the increase in neutral lipids, the amount of stored glycogen sharply decreased. A similar observation was made in the liver of Wistar rats fed with a choline-deficient diet [20], of Sprague-Dawley rats fed with a high-fat diet [51], and of Zucker* fa*/*fa* rats (a transgenic model of obesity) [52].

A further effect of the MCD diet was an increase in ROS production by hepatocytes throughout the whole lobule. As already mentioned, the main sources of ROS are complexes I and III of the mitochondria electron transport chain [35]. Enhanced *β*-oxidation of overloaded free fatty acids and uncoupling between electron transfer and energy production, often reported in NAFLD and NASH, favor abnormal electron leakage and overproduction of superoxide [16]. This was, in particular, described for mice fed with the MCD diet [19]. Furthermore, in the NAFLD and NASH settings, ROS may also be formed in peroxisomes, where very-long-chain fatty acids initiate *β*-oxidation [53], and in the smooth endoplasmic reticulum, where *ω*-oxidation of fatty acids mediated by cytochrome P450 2E1 (CYP 2E1) takes place [54]. Increased expression of CYP 2E1 in NAFLD was indeed reported [55–57].

In normal conditions, antioxidant systems control and inactivate ROS produced by mitochondria, with residual species serving signaling purposes [31, 58]. When in excess, ROS may react (a) with polyunsaturated fatty acid chains of phospholipids in mitochondria, in particular with those of cardiolipin in the inner membrane, fundamental for the functioning of the electron transfer chain [50, 59]; (b) with antioxidant enzymes (in particular with Mn-dependent SOD), reducing their activity; (c) with components of the electron transfer chain, increasing ROS production (“ROS-induced ROS release”) [59]; and (d) with mtDNA, inducing point mutation, DNA breaks, and decreased mtDNA content [21]. Such a vicious cycle of injury causes mitochondria dysfunction and impaired ATP production [21, 59]. Low hepatic ATP has often been reported for human fatty liver (e.g., [60–64]). The much lower ATP concentration of the liver of animals fed with the MCD diet observed in the present study might be explained by a similar process and is in keeping with published data concerning rats fed with MCD diet [10].

Protein carbonyl groups are considered as early markers of proteins altered by oxidative stress since they may be introduced in proteins by reaction of nucleophilic side chains of Cysteine, Histidine, and Lysine residues with aldehydes produced during lipid peroxidation (e.g., 4-hydroxy-2-nonenal and malondialdehyde, TBARS-positive) [38, 39]. Using the immunoreaction against DNP groups formed after derivatization of carbonyl groups with DNPH, and with respect to the controls, membrane staining of hepatocytes vanished from the 2nd week of MCD diet onwards and was instead concentrated and intense around large lipid vacuoles. This immunoreactivity is here ascribed to carbonyl groups in peroxidated proteins of the lipid droplets coat. The lobular distribution patterns of ROS and of peroxidated proteins along the 4-week period of MCD diet showed similar patterns suggesting a spatial correlation between the ROS production and protein oxidation.

The presence of peroxidated proteins surrounding lipid droplets observed in this study may be an important feature contributing to the dysfunction of hepatocytes in fatty liver. Triglyceride accumulation in lipid droplets is believed to be a mechanism of defense against cytotoxicity induced by free fatty acids [65]. Lipid droplets, present in normal and pathologic cell types, are dynamic organelles that may shrink or expand and that play roles that go far beyond lipid storage and catabolism [66, 67]. They contain a hydrophobic core of neutral and minimally charged lipids surrounded by a phospholipid monolayer [32]. Associated with or embedded in the phospholipid monolayer is a wide spectrum of proteins involved in the regulation of lipid turnover (e.g., perilipin, adipose differentiation-related protein, and tail-interacting protein of 47 kDa, collectively known as PAT), cell signaling, membrane trafficking, and metabolism of steroids, proteins, and carbohydrates [33, 68–73]. In particular, lipid droplet-associated proteins of the perilipin/PAT family were reported to be differentially expressed in hepatocyte steatosis, and that perilipin is expressed* de novo*, being a marker of chronic steatosis* in vivo* and* in vitro*, representing a final stage of lipid droplet maturation [71, 74]. Since antibodies against several lipid droplet-associated proteins are currently available, it would be worthwhile investigating the possible correlation between their expression and oxidative stress.

A correlation between liver steatosis and oxidative stress has been often reported in the literature (e.g., [20, 21]). Oxidative stress is believed to be the second hit leading to the development of steatohepatitis [5]. Most studies on oxidative stress in fatty liver were conducted with biochemical evaluation of parameters such as total glutathione (GSH) and thiobarbituric acid reactive substances (TBARs) or of antioxidant enzymes in tissue homogenates or in plasma. Very few reports were found in the literature regarding the use of a histochemical approach for documenting NAFLD-induced oxidative stress. In particular, our group had previously reported the* in situ* distribution of ROS in the liver of obese Zucker rats [75], which was similar to that found in the current research for rats fed with a 2-week MCD diet. More recently, we reported preliminary histochemical data concerning ROS, vitamin A, and peroxidated proteins distribution in the liver of rats fed with the MCD diet, related to the tissue autofluorescence spectrum [76]; the latter is an index of tissue metabolism and injury [77].

The immunohistochemical analysis of SOD2 revealed a change in protein expression pattern in hepatocytes along the 4-week period of the MCD diet that was similar, in particular in the last weeks, to that of ROS and peroxidated proteins patterns. In particular, large steatotic cells showed intense immunoreactivity in the thin rim of cytoplasm surrounding the lipid droplets. The correspondence between strong ROS production and intense SOD2 expression in the same areas of the lobule suggests an antioxidant attempt to compensate for the high ROS production. Such attempt, associated with the fact that GSH concentration was much lower than normal, might not be entirely successful, thus explaining the formation of peroxidated proteins in the same areas where the ROS production was high. The general trend towards decrease of SOD2 expression of liver homogenates in the last 2 weeks detected by Western Blotting is here ascribed to the much lower cytoplasm area occupied by the enzyme as hepatocytes acquire an adipocyte-like morphology.

As concerns the GSH concentration, though we found it to be not significantly different along the 4-week period of the diet, it is much lower with respect to the control. These data are in keeping with data reported in the literature concerning depletion of antioxidant defenses and in particular of GSH in NAFLD [21, 78], in particular induced by MCD diet [79]. The increase in TBARS along the period of the diet, detected in the present study, is in keeping with the high production of ROS, low concentration of GSH, and the moderately low SOD2 expression. It can be speculated that TBARS are the effectors leading to the oxidative alteration of proteins.

In conclusion, the histochemical approach herewith illustrated and whose main results are summarized in Table 1 complements and supports biochemical data regarding the insurgence of oxidative stress induced by a MCD diet. It allowed visualizing* in situ* the induced alteration of hepatocyte morphology, glycogen stores, lipid droplet behavior, ROS production, and expression of the antioxidant enzyme SOD2. In particular, it points towards chemical modifications of proteins involved in lipid maintenance and metabolism.

## Figures and Tables

**Table 1:** Summary of the relevant observations regarding metabolic and oxidative stress alterations induced by a 1–4-week period of MCD diet, with respect to control diet.

Parameter	Diet-induced modifications
Glycogen content	Sharp decrease

Neutral lipid content	Progressive increase

Reactive Oxygen Species	Progressive increase especially surrounding lipid droplets

Peroxidated proteins	Progressive increase especially surrounding lipid droplets

Superoxide Dismutase 2 expression	Concentrated in thin rim of cytoplasm surrounding lipid droplets; trend towards decreased expression in the last 2 weeks

## Abbreviations

DNP: Dinitrophenyl
GSH: Glutathione
LD: Lipid droplet
MCD: Methionine- and choline-deficient
NAFLD: Nonalcoholic fatty liver disease
NASH: Nonalcoholic steatohepatitis
ROS: Reactive Oxygen Species
PAT: Perilipin, adipose differentiation-related protein, and tail-interacting protein of 47 kDa
SOD: Superoxide Dismutase
VLDLs: Very-low-density lipoproteins
SAM: S-Adenosylmethionine
TBARS: Thiobarbituric acid reactive substances.
